# Dental anomaly detection using intraoral photos via deep learning

**DOI:** 10.1038/s41598-022-15788-1

**Published:** 2022-07-08

**Authors:** Ronilo Ragodos, Tong Wang, Carmencita Padilla, Jacqueline T. Hecht, Fernando A. Poletta, Iêda M. Orioli, Carmen J. Buxó, Azeez Butali, Consuelo Valencia-Ramirez, Claudia Restrepo Muñeton, George L. Wehby, Seth M. Weinberg, Mary L. Marazita, Lina M. Moreno Uribe, Brian J. Howe

**Affiliations:** 1 Department of Management Sciences, Tippie College of Business, University of Iowa, Iowa City, IA USA; 2 Department of Pediatrics, College of Medicine, University of the Philippines, Manila, Philippines; 3 Department of Pediatrics, University of Texas Health Science Center at Houston, Houston, TX USA; 4 ECLAMC at Center for Medical Education and Clinical Research, CEMIC-CONICET, Buenos Aires, Argentina; 5 ECLAMC at Department of Genetics, Institute of Biology, Federal University of Rio de Janeiro, Rio de Janeiro, Brazil; 6 Dental and Craniofacial Genomics Core, School of Dental Medicine, University of Puerto Rico, San Juan, PR USA; 7 Department of Oral Pathology, Radiology, and Medicine, University of Iowa, Iowa City, IA USA; 8 The Iowa Institute for Oral Health Research, College of Dentistry, University of Iowa, Iowa City, IA USA; 9 Clinica Noel, Medellín, Colombia; 10 Department of Health Management and Policy, College of Public Health, University of Iowa, Iowa City, IA USA; 11 Center for Craniofacial and Dental Genetics, School of Dental Medicine, University of Pittsburgh, Pittsburgh, PA USA; 12 Department of Orthodontics, College of Dentistry, University of Iowa, Iowa City, IA USA; 13 Department of Family Dentistry, College of Dentistry, University of Iowa, Iowa City, IA 52242 USA

**Keywords:** Machine learning, Paediatric research, Dentistry, Diagnosis

## Abstract

Children with orofacial clefting (OFC) present with a wide range of dental anomalies. Identifying these anomalies is vital to understand their etiology and to discern the complex phenotypic spectrum of OFC. Such anomalies are currently identified using intra-oral exams by dentists, a costly and time-consuming process. We claim that automating the process of anomaly detection using deep neural networks (DNNs) could increase efficiency and provide reliable anomaly detection while potentially increasing the speed of research discovery. This study characterizes the use of` DNNs to identify dental anomalies by training a DNN model using intraoral photographs from the largest international cohort to date of children with nonsyndromic OFC and controls (OFC1). In this project, the intraoral images were submitted to a Convolutional Neural Network model to perform multi-label multi-class classification of 10 dental anomalies. The network predicts whether an individual exhibits any of the 10 anomalies and can do so significantly faster than a human rater can. For all but three anomalies, F1 scores suggest that our model performs competitively at anomaly detection when compared to a dentist with 8 years of clinical experience. In addition, we use saliency maps to provide a post-hoc interpretation for our model’s predictions. This enables dentists to examine and verify our model’s predictions.

## Introduction

Individuals with orofacial clefting (OFC) present with a wide range of complex dental anomalies that affect tooth size, shape, structure, number, symmetry, and position, thus increasing phenotypic complexity and dental morbidity in affected individuals. Amongst these anomalies, the most common ten types include hypoplasia, hyopcalcification, agenesis, mammalons, microdontia, supernumerary teeth, impacted teeth, tooth rotations, and displacements. Although dental anomalies may often appear in the general population (up to 22% in the primary and 47% in the permanent dentition), their occurrence in individuals affected with overt clefts is much higher (up to 45% in the primary and 61% in the permanent dentition) and their etiology remains unknow^1–6^. Accurate and efficient identification of dental anomalies is vital to understanding their etiology, management and prevention. Specifically, the development of methods for large-scale screening of dental anomalies in human populations with high accuracy and effectiveness will largely increase the precision of association or causality estimates of genetic and environmental effects on such anomalies. In this work, we identify inefficiencies in the screening process and propose a deep learning based method to address them.

Currently, in-person dental exams, review of radiographs, and/or intraoral photographs are used to identify and document dental anomalies. However, these methods are labor-intensive, requiring training and careful calibration and are very time consuming, particularly for large samples, and thus can in turn slow down the speed of discovery. For instance, in our previous studies with large data, it took 1 year for a human rater to score over 30,000 intraoral images (IOPs) in 4084 subjects^6^. In addition, in the current human rater method, bias and errors in identification can occur and thus inter and intra-rater reliabilities of the dental anomaly data acquired are important aspects of data integrity that must be considered. These challenges are compounded in multicenter studies since an increase in the number of raters is required to complete data collection efficiently. Machine learning methods such as deep learning may be a promising solution to score large data sets objectively, reliably, and efficiently. While it takes years to train a human rater, in only takes hours to train a machine learning model. We also claim that in the long run, using machine rather than human labor saves significant time in scoring and can increase discovery speed.

In recent years, convolutional neural networks (CNNs) have become a state-of-the-art solution for image classification and have been successfully applied to dentistry^7^. CNNs are a particular type of Deep Neural Network (DNN). A recent survey^7^ reported on about 30 published papers (as of April 2021) in the intersection of deep learning and dentistry. Examples include using CNNs to detect periapical lesions, dental caries, and odontogenic cystic lesions. However, it indicates that only very few publications^8,9^ use digital camera photos as input data. The majority of existing work trained a CNN model using medical images such as radiographs or computed tomography scans that must be obtained by medical devices and are costly for patients. Use of digital cameras as opposed to specialized equipment both simplifies the data collection process and saves on hardware costs. The improved obtainability of the image makes our method accessible for patients and easier to use.

A potential challenge for deep learning is that, in order to perform well, deep learning methods rely heavily on the amount of available training data. The models need to “see” enough examples to fit the large number of parameters. Previous dental literature used relatively small data sets of, at most, a few thousand images^7–9^. Our data set presents a unique opportunity to implement a deep learning method by having access to a sample that is orders of magnitude larger than previous research, collected from the largest international cohort, to-date, of subjects with OFC and controls.

Besides having a relatively large training dataset, our model also benefits from transfer learning (TL). The technique of TL starts with acquiring a trained CNN image classifier developed using a large number of images. The next step is to re-train the classifier on a new dataset but usually with the weights of the first few layers kept unchanged (frozen). Transfer learning can improve the predictive performance of a CNN because the low and mid-level feature transformation is very similar across different image classification tasks regardless of the target variable. Thus, our model can effectively “borrow” knowledge from existing state-of-the-art models. The TL technique can mitigate the lack of data problem as it uses information from other sources to build the model. We have identified only 13 publications in the dental image classification literature since 2017 that have utilized TL^8–18^.

**Figure 1:**
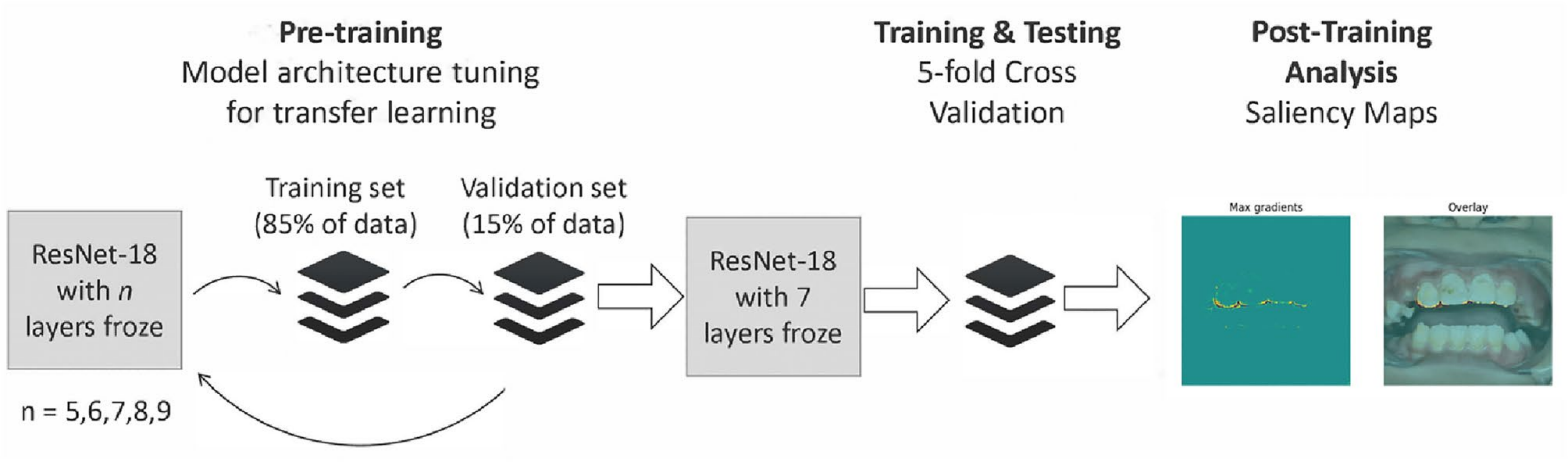
Workflow of data analysis. The entire workflow consists of three steps. In step 1, we tune the number of layers to freeze in order to do TL optimally. Our experiments show that when freezing 7 layers, our model achieved the best predictive performance. We then test the model using a fivefold grouped cross-validation. Finally, for each input photo and the corresponding model prediction, we generate a saliency map for each anomaly (regardless of presence in the photo).

While deep learning models can achieve highly accurate predictive performance, their “black-box” nature has been criticized for hindering human understanding^19^, especially in medical applications. Therefore, in addition to classifying the presence of anomalies, we also aim to provide interpretable post-hoc explanations for why the model makes such a diagnosis, by showing users which part of the image the model has focused on for a given intraoral photo. To do that, we generate a saliency map highlighting the area that is considered most important for the CNNs output. Figure 1 shows the workflow of our data analysis.

In summary, the goal of the present study is to use deep learning (CNN and TL) to classify dental anomalies (agenesis, hypoplasia, hypocalcification, impacted teeth, incisal fissures, mammalons, microdontia, supernumerary teeth, and tooth rotation and displacement) for an input IOP and be comparable yet highly efficient compared to an expert human rater. To enable human interpretation, we generate saliency maps to provide explanations for how the CNN classifies images as having or not having dental anomalies, allowing verification of the predictions when using our method in practice.

The ability to do image classification with intraoral photos is foundational to facilitating personalized dentistry. In such a system, the practitioner would be able to upload intraoral photos and get diagnostic results via a trained neural network as well as explanations for the anomaly identification via a saliency map, thereby aiding the dentist in their diagnostic abilities and expediting clinical visits. Automating the process of dental anomaly classification using DNNs could also increase reliability, reproducibility, and the speed of anomaly classification. If a DNN is equal to or superior to human-raters while being significantly faster and more objective, the DNN-based image processing framework has the potential to revolutionize data collection methods and increase the speed of research discovery in orofacial biology and beyond.

## Methods

### Dataset

The study consisted of 38,486 intraoral photographs in 4,084 subjects (765 with OFC and 3319 control subjects). Intraoral photos and associated anomaly data was utilized from a previous study (OFC1)^6^ from multiple sites in the United States and internationally. This study was reviewed by the Internal review board (IRB) at the University of Iowa and determined to be exempt from IRB review. All methods were carried out in accordance with relevant guidelines and regulations. Informed consent was obtained from each subject or their legal guardian(s) as part of the original study (OFC1). For each subject, a series of 6–10 intraoral photographs were made to fully display the entire oral cavity. Information corresponding to each subject, such as cleft status and the presence of various anomalies on each tooth, was logged into the OFC1 database. Each subject’s photo set was evaluated and scored for dental anomalies using a paper form developed for this use (Supplementary Fig. S1). The rater, BJH, was a dentist with 8 years of clinical experience and was calibrated against two more experienced dentists for identification of dental anomalies on a small validation dataset prior to OFC1 data collection. Intra-rater reliability for BJH was 100% agreement with kappa = 0.95. Inter-rater reliability between all three raters was between 97.1 and 97.3% agreement with kappa = 0.91–0.93. After calibration, BJH scored all subjects and photos, becoming the ground truth. The training data for our CNN are constructed from OFC1 as follows. Given a photo, we assign the label for that photo a length 10 binary vector where each of the 10 indices corresponds to one of the 10 anomaly types we consider. The vector is 1 at an index if the patient in the photo has the corresponding anomaly *on any tooth*, and 0 otherwise. For additional details used in the collection of data, see the supplementary material.

### Model architecture

We adopted state-of-the-art methods for image classification by using transfer learning with a popular CNN architecture. There are a few classic resources in which details on CNNs may be found^8^. In addition to a CNN, we adopted TL^20^, which utilizes a pre-existing CNN that has been trained on a very large dataset of photos and adapted it to our task. This allows us to further boost the predictive performance and save a substantial amount of computation time.

The pre-trained CNN we have chosen is ResNet-18^21^. It is an 18-layer CNN that has been trained using fourteen million images from the ImageNet database^22^. We experimented with freezing a different number of layers while leaving the rest of the layers trainable to adapt the model to our dental anomaly classification task. Results show that the best performance was achieved when freezing the first 7 layers. Since each study subject can have multiple anomalies, we designed a multi-label multi-class output layer with 10 nodes, each representing a type of anomaly. Each node then produces a probability for an input to have the corresponding anomaly. Our model uses raw pixel data from intraoral photos and preprocesses them using the standard ImageNet procedure. For additional information on network architecture and methods, see the supplementary material.

### Training and evaluation

We tasked our CNN with making accurate classifications of dental anomaly presence in each photo, judging it by means of accuracy, F1, ROC/AUC, and precision/recall metrics. The dataset used to train, test and validate the model consists of the 38,486 photos in OFC1. We conducted a group fivefold cross validation of our model. This cross-validation variant splits the data into five subsets such that each subset consists of 20% of the data. It differs from standard cross validation in that it splits data by patients, which ensures that patients are not represented in more than one fold, and each fold represents approximately the same number of patients. In each fold, four subsets of them are combined into a training set while the remaining is the testing set. This is done five times such that each subset is used as a test set once. We set the batch size to be 512 images, number of epochs to be 1000, and the initial learning rate to be 1.34E-6. We use the AMSGrad^23^ variant of the AdamW optimizer in PyTorch^24^. For each epoch, the model takes in a batch of images and uses the AdamW optimizer to optimize the parameters in the fully connected layer to minimize the multi-class dice loss between the outputs and the true values. We found this loss function to yield better results than other means of tackling class imbalance, including using weighted binary cross entropy loss or focal loss. Using the principle of early stopping, if the model sees that in 60 consecutive epochs the validation loss has not decreased, it will cease training early to prevent overfitting. Further details appear in the supplementary material.

### Saliency maps

To provide an interpretable explanation to the results provided by our CNN, we generated a saliency map for each output, to show what regions of an input image were considered important by the model to produce the corresponding classification^25^. One may consider the outputs of a CNN as a vector of differentiable probability functions. A saliency map is a heat-map where the intensity of each pixel is calculated by taking the gradient of the functions produced by the CNN for each of the anomalies. The value represents the contribution of the corresponding pixel of an input image to a class score^26^. The higher the value, the more important the pixel is for the CNN model’s classification decision. These gradients are computed per color channel of the input image. To obtain a heat map of gradients across an image, the max gradient can be used over each color channel. In our max gradient saliency maps, the color of each pixel ranges from blue (cold) to red (hot) depending on how big the max gradient was for that pixel. The saliency map allows for interpretability of the image and confirms that the CNN model is reliably identifying the correct anomalies.

## Results

### Predictive performance

We evaluated our model using the test sets of each of the five folds for the tasks of classifying whether or not each patient has each anomaly. We report F1, ROC/AUC, precision, and sensitivity for each anomaly for our model in Table 1. For our model, the mean F1 score from the 5-folds for each anomaly, which is a reflection of the specificity and precision of the model, ranged from 0.437 to 0.561, with hypoplasia having the highest F1 score and hypocalcification having lowest F1 scores (0.561 and 0.437 respectively). The median AUC for each anomaly ranged from 0.683 to 0.872 with displaced teeth having a lowest AUC (0.66). The model had special difficulty in classifying incisal fissures and hypoplasia. The frequency of anomalies per image can be found in Supplementary Table S1.

**Table 1:** Model metrics.

Anomaly	F1	Precision	Recall	ROC AUC
Mammalons	0.506 ± 0.077	0.482 ± 0.026	0.637 ± 0.205	0.633 ± 0.122
Impacted	0.540 ± 0.099	0.486 ± 0.163	0.774 ± 0.258	0.677 ± 0.125
Hypoplasia	0.561 ± 0.086	0.531 ± 0.205	0.806 ± 0.272	0.708 ± 0.144
Incisal Fissure	0.531 ± 0.139	0.496 ± 0.240	0.787 ± 0.212	0.651 ± 0.138
Hypocalcification	0.437 ± 0.059	0.397 ± 0.290	0.619 ± 0.229	0.590 ± 0.089
Displaced	0.482 ± 0.122	0.374 ± 0.115	0.729 ± 0.182	0.682 ± 0.084
Microdontia	0.517 ± 0.078	0.430 ± 0.140	0.670 ± 0.312	0.685 ± 0.080
Supernumerary	0.478 ± 0.101	0.534 ± 0.059	0.746 ± 0.252	0.571 ± 0.123
Rotation	0.443 ± 0.097	0.388 ± 0.154	0.868 ± 0.184	0.562 ± 0.100
Agenesis	0.544 ± 0.093	0.533 ± 0.231	0.728 ± 0.252	0.678 ± 0.083

Results given are the mean result of all fivefolds with the standard deviation.

### Comparison of CNN with human baseline

In addition to the above evaluate, we compare our model against a human rater. On a subset of 30 patients from OFC1, we record BJH’s *pre-calibration* performance for the tasks of detection of each anomaly in Table 2. (Note that the data used to train and evaluate the model were labeled after BJH was calibrated) BJH classified whether or not each individual had each anomaly by examining all of their IOPs (this differs from our model, which classifies anomaly presence in each photo separately)*.* LMU, a more experienced dentist, also classified the anomaly presence in the 30 patients. We used BJH’s results as a ground truth to evaluate LMU’s pre-calibration F1, precision, recall, sensitivity, and specificity metrics for each anomaly. F1 scores in Table 2 are recorded as 0 if LMU make no correct predictions. They are recorded as N/A if there were neither positive ground truth labels nor predictions of the positive label. Incisal fissures has a precision of N/A because LMU had neither true positives nor false positives. Supernumerary has a recall of N/A because LMU had neither true positives nor false negatives.

**Table 2:** LMU pre-calibration metrics.

Anomaly	F1	Precision	Recall
Mammalons	0.857	1.000	0.750
Impacted	N/A	0.000	0.000
Hypoplasia	0.667	0.500	1.000
Incisal Fissure	0.000	N/A	0.000
Hypocalcification	0.400	1.000	0.250
Displaced	0.246	0.750	0.750
Microdontia	N/A	0.000	0.000
Supernumerary	0.000	0.000	N/A
Rotation	0.963	1.000	0.929
Agenesis	0.000	0.000	0.000

We use LMU’s pre-calibration performance against BJH to get an idea of how our model compares with an actual dentist. We find that our model compares favorably to LMU. Although LMU’s F1 scores for mammalons (0.857) , hypoplasia (0.667), and rotation (0.963) are higher than the model’s (0.506, 0.561, and 0.443 respectively), BJH’s F1 scores are lower for the remaining anomalies. See Tables 1 and 2. We also found the difference in time required, on average, to classify anomaly presence to be significant. In this study, the training routines generally took on the order of 12 h, while BJH has accumulated experience over 8 years of clinical experience. The test step took approximately 3 min for 7,697 photos, a rate of approximately 40 photos per second. Thus, if the model were to classify all 38,486 photos, it will need approximately 16 min to complete the task whereas it took a human-rater one year^6^.

### Post-hoc interpretability via saliency

**Figure 2:**
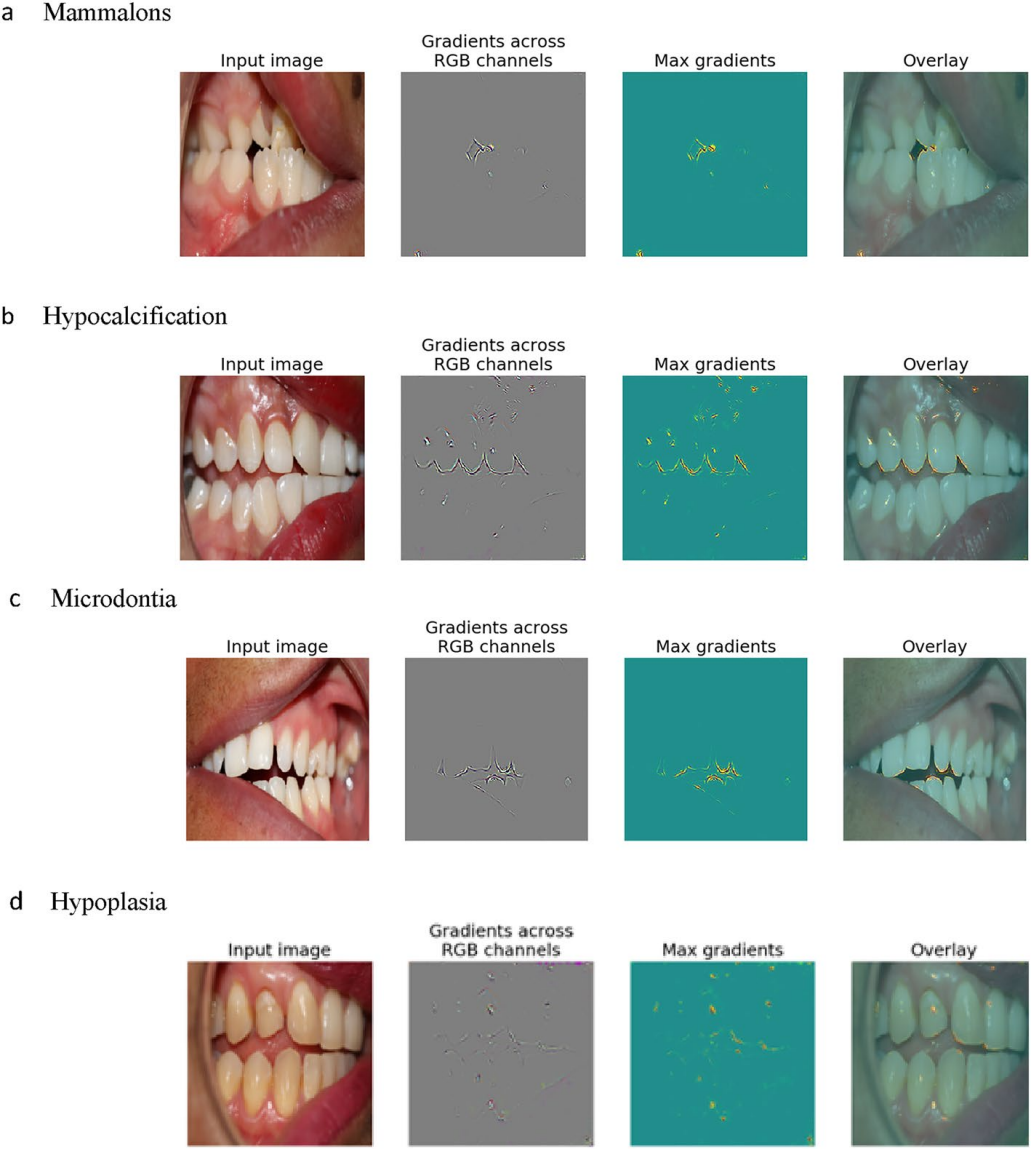
Saliency maps. *Note*: Overlay is the input image overlaid with the gradients. These are representative examples of anomalies depicting what the algorithm saw when making correct predictions of mammalons, hypocalcification, microdontia, and hypoplasia.

To enable human understanding, we generated saliency maps to show important image regions when our CNN (correctly) predicts each of the 10 considered anomalies. See Fig. 2 for examples. For example, in Fig. 2a the saliency map highlights the incisal edge of the mandibular incisors, indicating that the CNN is recognizing the relevant area where mammalons occur and in Fig. 2b reveals hypocalcification on the maxillary right canine and the CNN highlighted the incisal edge areas. In addition, we also examined saliency maps for *incorrect* predictions, which is particularly important since if domain experts understand why the model makes a mistake, then they know when not to trust a model. We found that when a model makes a mistake, it often looks at non-relevant area of the images such as gingiva, buccal mucosa, or space between teeth. We also found that orthodontic appliances such as arch wires, brackets, and fixed retainers, are difficult for the CNN to ignore and could mislead the CNN. Orthodontic appliances can obscure dental anomalies for the CNN and human rater alike, thus this limitation could be applied to both. We also found that the CNN has difficulty with blurry or unfocused intraoral photos or those that depict a narrow or small field of view. We randomly sampled 100 mis-classified samples (10 for each anomaly type) and found that 21 had braces in them. 34 of them showed only a narrow region of the mouth. 4 of them were completely blurry. See Supplementary Fig. 3 for examples of saliency maps where the highly activated regions do not correspond with the actual locations of the anomalies.

## Discussion

The use of image classification algorithms such as TL with CNNs has become increasingly popular in the past few years. Our findings suggest great potential in use of CNN-based image classification for quickly identifying dental anomalies from intraoral photos. The ability to produce saliency maps makes our method interpretable and provides insight into the model’s reasoning. Our method not only performs dental anomaly classification but can also show where in the mouth the CNN “looks” to make its decision. Clinicians and researchers can, therefore, consult the saliency map and verify whether the CNN model is making classifications that are consistent with the location and development of such anomalies. This can give additional confidence for clinicians and researchers using this model and can provide educational benefits for students and less experienced clinicians. In addition, since our method can work with intraoral photos taken by standard cameras, it is more accessible than other DNN based models that work with X-rays or CT-scans.

In the current study we used ResNet-18 as the pre-trained CNN model^21^ for TL. This CNN was chosen over other models due to its low runtime and high accuracy when compared to other popular architectures on the ImageNet benchmark. ResNet-18 is a popular open-source network architecture, so theoretically if independent clinics were using our training methodology with separate private datasets, they could share model weights or training gradients in order to benefit from each-other’s data without sharing their data.

The dataset was originally scored for dental anomalies, by one person after calibration6 (also supplementary material) and took approximately one year of full-time work to score all 4,084 subjects and their respective 38,486 intraoral images. In the current study, the CNN would be able to identify the dental anomalies in the same number of photographs in approximately 16 min with F1 scores ranging from 0.32 to 0.989. Our results suggest that our model is able to perform at a similar level as that of a dentist with 8 years of clinical experience in the anomaly detection tasks. See additional metrics in Table 1. We found examples of both classification agreement and disagreement, for example where the model correctly predicted hypoplasia while the human rater did not, see Supplementary Fig. S2. This highlights the error that can occur from eye fatigue or human error that does not occur in computers.

To be successful at image classification tasks, a CNN needs to be trained on a very large number of examples in order to learn good feature representations from the input images. The size of the training examples has a direct impact on the overall model accuracy. The current data set is the largest international cohorts of intraoral photos of controls and subjects with OFC, with 38,486 images. For multi-label multi-class image classification task, this is still considered small. However, we were able to achieve reasonable F1 scores (0.437–0.561) using the technique of transfer learning. To continue to improve and test the accuracy of this model, additional intraoral photographs will be needed. A second intraoral data set has been scored and will be used to further test and improve this algorithm to see if it can equal or outperform human raters on every dental anomaly. We used a separate sample of data to get an estimate of human performance with respect to the F1, precision, and recall metrics. For all but three types of anomalies, our model’s F1 scores exceeded those of the human baseline.

Data imbalance between subjects with OFC and controls is a limitation of this study as subjects with OFC have a higher incidence of certain dental anomalies. To address this, we tested different loss functions that are supposed to be robust to data imbalance. We tested weighted binary cross entropy, multi-class dice loss, and focal loss. The multi-class dice loss proved to yield the best performance. Another limitation of the current algorithm is that it does not give dental anomaly data per tooth, but whether any of the anomalies are present in the photograph per subject. Future work is needed, and is currently underway, for the CNN to identify each tooth in each photo and the associated anomalies.

In examining the saliency maps generated by the model, we found that orthodontic appliances such as arch wires, brackets, and fixed retainers, are difficult for the CNN to ignore and is a limitation of the study. Orthodontic appliances can obscure dental anomalies for the CNN and human rater alike, thus it is a limitation for providers and the CNN. Blurry or unfocused intraoral photos or those that depict a narrow or small field of view are also a limitation of this study. This limitation can be solved by providing more high quality photos to the model.

This algorithm also has the potential to be a second rater to calibrate against or even a replacement for the rater with further validation, which will increase the speed of data collection and analysis while saving cost. This method could be used in the field when intraoral-photos are made, uploaded, run through the algorithm and the results transmitted to the principal investigator from sites around the world, thus the person-hours needed for dental anomaly classification could decrease significantly assisting oral health research around the globe. Another possible application would be a dental phenotype-to-gene or tooth-to-gene, where the CNN identifies the dental anomalies per subject and link this with an available genetic database to produce possible genes linked to the identified dental anomalies, similar to FACE2GENE (FDNA, Boston, MA).

## Conclusion

In this work we proposed to use ResNet-18 and transfer learning to detect the presence of 10 dental anomalies using Intra-Oral Photos (IOPs) from standard cameras as inputs. In isolation, we found our method to obtain fairly good classification accuracy. When compared to human dentists, our method boasts significantly faster classification speed and competitive accuracy. To mimic the way human dentists can point out where they looked to recognize the presence of a dental anomaly, we used saliency maps to show where our model was looking when making predictions, which enable human dentists to understand the reasoning of our model.

Our algorithm has the potential to change how dental anomalies are scored and thus how dental anomaly phenotypes are identified in populations. It can greatly increase the speed of discovery by taking a task that potentially can take years, with a large data set similar to the current one, to taking a couple of hours. Using it instead of or in tandem with human raters would lower long-term costs for identification of dental anomalies. In the future, for image analysis of dental anomalies, data collection and analysis may take place simultaneously, transmitted to the research team for the findings to be interpreted via a secure website, which is under development. Further research is needed in this exciting area of dental research.

## Supplementary Information

Supplementary Information.

## Data Availability

Data is available upon request for mutual collaboration.

## Code availability

The code used in this study is available here https://github.com/rrags/DentalAnomalyDetector.
