# Author Correction: Dental anomaly detection using intraoral photos via deep learning

**DOI:** 10.1038/s41598-022-17668-0

**Published:** 2022-08-08

**Authors:** Ronilo Ragodos, Tong Wang, Carmencita Padilla, Jacqueline T. Hecht, Fernando A. Poletta, Iêda M. Orioli, Carmen J. Buxó, Azeez Butali, Consuelo Valencia-Ramirez, Claudia Restrepo Muñeton, George L. Wehby, Seth M. Weinberg, Mary L. Marazita, Lina M. Moreno Uribe, Brian J. Howe

**Affiliations:** 1 Department of Management Sciences, Tippie College of Business, University of Iowa, Iowa City, IA USA; 2 Department of Pediatrics, College of Medicine, University of the Philippines, Manila, Philippines; 3 Department of Pediatrics, University of Texas Health Science Center at Houston, Houston, TX USA; 4 ECLAMC at Center for Medical Education and Clinical Research, CEMIC-CONICET, Buenos Aires, Argentina; 5 ECLAMC at Department of Genetics, Institute of Biology, Federal University of Rio de Janeiro, Rio de Janeiro, Brazil; 6 Dental and Craniofacial Genomics Core, School of Dental Medicine, University of Puerto Rico, San Juan, PR USA; 7 Department of Oral Pathology, Radiology, and Medicine, University of Iowa, Iowa City, IA USA; 8 The Iowa Institute for Oral Health Research, College of Dentistry, University of Iowa, Iowa City, IA USA; 9 Clinica Noel, Medellín, Colombia; 10 Department of Health Management and Policy, College of Public Health, University of Iowa, Iowa City, IA USA; 11 Center for Craniofacial and Dental Genetics, School of Dental Medicine, University of Pittsburgh, Pittsburgh, PA USA; 12 Department of Orthodontics, College of Dentistry, University of Iowa, Iowa City, IA USA; 13 Department of Family Dentistry, College of Dentistry, University of Iowa, Iowa City, IA 52242 USA

Correction to: *Scientific Reports* https://doi.org/10.1038/s41598-022-15788-1, published online 08 July 2022

In the original version of this Article Ronilo Ragodos, Tong Wang and Brian J. Howe were omitted as equally contributing authors.

Tong Wang was omitted as an additional corresponding author. Correspondence and requests for materials should also be addressed to tong-wang@uiowa.edu.

In addition, the Author Contributions section in this Article was incorrect.

“A.B., C.P., J.T.H., F.A.P., I.M.O., C.J.B., C.V.-R., C.R.M., and G.L.W. contributed to conception, design, and data acquisition, drafted and critically revised the manuscript; R.R. and T.W., contributed to conception, design, analysis, and data interpretation, drafted and critically revised the manuscript; B.J.H., S.M.W., M.L.M., and L.M.M.-U. contributed to conception, design, data acquisition, analysis, and interpretation, drafted and critically revised the manuscript. All authors gave final approval and agree to be accountable for all aspects of the work.”

now reads:

“A.B., C.P., J.T.H., F.A.P., I.M.O., C.J.B., C.V.-R., C.R.M., and G.L.W. contributed to conception, design, and data acquisition, drafted and critically revised the manuscript; R.R. and T.W., contributed to conception, design, machine learning methods, analysis, and data interpretation, drafted and critically revised the manuscript; B.J.H., S.M.W., M.L.M., and L.M.M.-U. contributed to conception, design, analysis, and data interpretation, drafted and critically revised the manuscript. All authors gave final approval and agree to be accountable for all aspects of the work.”

The original Article has been corrected.

