# Supplementary material for: Dental anomaly detection using intraoral photos via deep learning
Source: Sci Rep. 2022 Jul 8;12:11577. doi: 10.1038/s41598-022-15788-1 (PMC9270352; doi:10.1038/s41598-022-15788-1)
Supplement: Supplementary file 1 — Supplementary Information. [file 41598_2022_15788_MOESM1_ESM.docx]

Supplementary Material

**Methodology**

**Dataset**

Subjects were recruited from multiple sites in the United States (Iowa, Pennsylvania, Puerto Rico, and Texas) and internationally (Colombia, Guatemala, Hungary, and the Philippines). IRB approval was obtained at each site. The sample included subjects in family units with nonsyndromic orofacial clefting (OFC) (case probands) (cleft lip and palate, cleft lip only, and cleft palate only) and unaffected family members (parents and siblings) and control subjects including control probands and control relatives (parents and siblings). Exclusion criteria for controls included a positive family history of OFC or syndromes and a history of facial trauma or surgery. Edentulous individuals were excluded in all subject groups.

Questionnaires recording dental history were collected on all patients. All subjects used in the current study had intraoral photos taken. Sites were provided with cameras (Cannon EF 100mm f/2.8 macro USM lens, Cannon macro MR-14EX ring flash) and appropriate supplies for intraoral photo collection. Prior to photographs, all removable appliances/prostheses were removed. A minimum of six photographs were taken per subject to appropriately display the entire oral cavity (right and left lateral, anterior closed, maxillary and mandibular arches). The photo rater was blinded to gender, ethnicity, cleft status (in absence of obvious clefting), and family relations.

Calibration and training for intraoral photos was performed at the University of Pittsburgh for all the different sites prior to the start of data collection. The photo rater, BJH, was calibrated against two experienced dentists in the field of orofacial clefting, LMU and ARV. Thirty subjects, randomly chosen, were used for calibration. During the calibration stage, when the raters disagreed, the raters would review the subject and anomaly in question and come to an agreement regarding the rating of that anomaly on that subject and specific tooth. Each subject was rated two times by each rater (BJH, LMU, ARV) with at least 2 weeks between ratings. Intra-rater reliability for BJH was 100% agreement with kappa = 0.95. Inter-rater reliability between all 3 raters ranged from 97.1% - 97.3% agreement with *kappa* = 0.91-0.93.

Not all images in the data set were intraoral photos, such as photos of ID numbers for identification purposes or full facial photos, these images were removed from the data folders, for a total of 38,486 photos used in the current study.

**Convolutional Neural Network Architecture**

In addition to the Convolutional Neural Network (CNN), we utilized Transfer Learning. The idea behind transfer learning is that the feature learning through the hidden layers from the original image to the last convolutional layer, is similar across different images; be it animals, cars, or teeth. For example, the lower-level features often represent lines and strokes, the mid-level features often represent corners and circles, and the high-level features form more complicated patterns that are domain specific.

We perform transfer learning using Resnet-18. This particular network architecture was chosen as we believe it had the best tradeoff between runtime and accuracy, per baselines comparisons listed here: <https://github.com/jcjohnson/cnn-benchmarks>. We choose to freeze the first 7 layers of the Resnet-18 model since we found this configuration to yield higher F1 scores in a single fold experiment, than models where the first 5, 6, 7, 8, or 9 layers were frozen.

Labels for the training, testing, and validation images come from an OFC1 database generated spreadsheet. Correspondence between images and rows of the aforementioned spreadsheet come from an encoded patient identifier, which is present in the names of our intraoral photos and in the spreadsheet. The model then outputs class probabilities for each anomaly, which are transformed into binary class labels using thresholding. That is, if an output corresponding to an anomaly exceeds the threshold value, we assign the label 1 to signify presence of that anomaly. Otherwise, we assign 0. The output is then a vector of length ten whose entries correspond to each dental anomaly we consider.

**Training Procedure**

To evaluate our model, we do a grouped 5-fold cross validation. The experiments were run on heterogeneous cluster hardware. Each experiment used 16 CPU cores for data loading and trained the neural networks on either a RTX 2080Ti or Tesla P100. The folds were sampled randomly because we found that stratifying them to ensure identical label distributions significantly harmed the performance of the classifier. Each experiment begins by initializing a Resnet-18 model with its first 7 layers frozen. Then, on each fold, 20% of data is split from the training set to be used as a validation set for early stopping the training step.

The dataset is imbalanced in that most anomaly types have many more instances of the negative class than the positive class. To address this, we first experimented with reweighting and resampling the data. Reweighting refers to the strategy of weighting the terms of the loss function so that the loss is broken down by class and the class-wise loss is weighted in a manner proportional to the ratio of negative to positive examples in each class. That is, the more imbalanced the distribution of labels for a class is, the more the classifier will be penalized for misclassifying that class. In our specific case, the loss function was binary cross entropy. Resampling in our work refers to using a weighted random sampler for the training, validation, and testing sets that draws samples (with replacement) proportionally to the sum of the total numbers of times that anomalies present in a sample appear in the entire dataset. We also experimented with using loss functions which are claimed in the literature to be more robust to data imbalance than the standard binary cross entropy loss. In particular, we tried the focal loss and dice loss. In our work, we found using the dice loss and no resampling or reweighting to give the best performance.

Although we are doing grouped 5-fold cross validation, we reserve a 20% validation set from each training set for the sake of early stopping. The stopping rule used is that if, during the training procedure, the loss does not decrease over the course of 60 consecutive epochs, the training procedure ends prematurely. Otherwise, the training proceeds for 150 epochs. After this, during the testing phase, we evaluate the classifier on the test set (20% of data) in each fold and compute the class-wise confusion matrices, F1 scores, sensitivity, specificity, ROC curves, and the associated AUCs. We used consecutive 1-fold experiments to tune the classifier prediction thresholds for each class. These are computed as the points on the ROC curves that maximize the F1 score for the problem of classifying each image correctly for each class. Using these thresholds, we then pose the problem of classifying each patient correctly. To do this, for each class, we set the probability of a patient having a positive class label as the proportion of positively labeled images of that patient. A second set of thresholds is obtained to maximize the F1 scores for the problems of classifying whether each patient has each anomaly.

**Supplementary Figure 1: Intraoral Photograph Evaluation Form Example**

**Supplementary Figure 2: Saliency maps from instances when model made correct prediction but human did not**

1. Hypoplasia


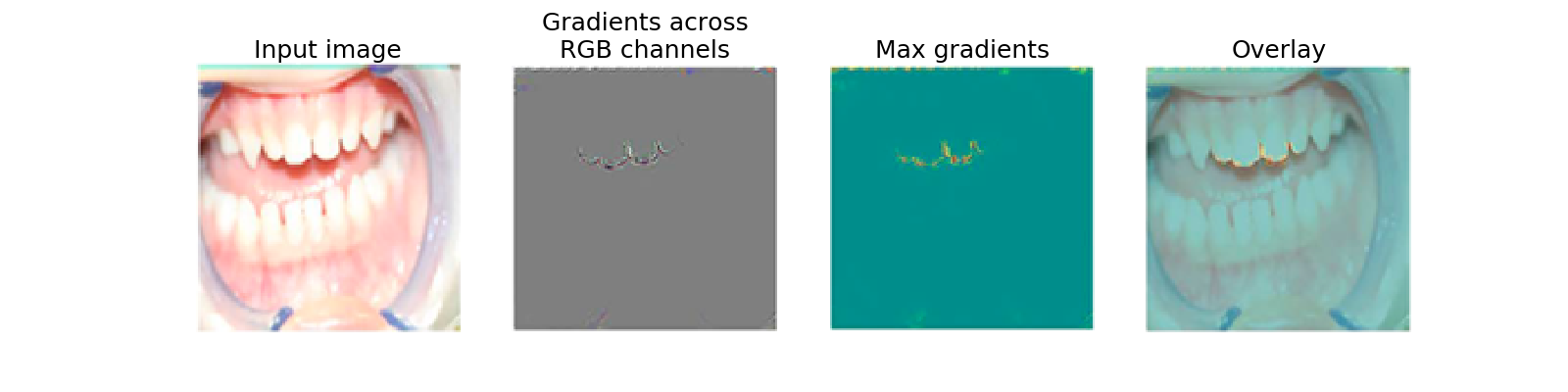


1. Hypoplasia


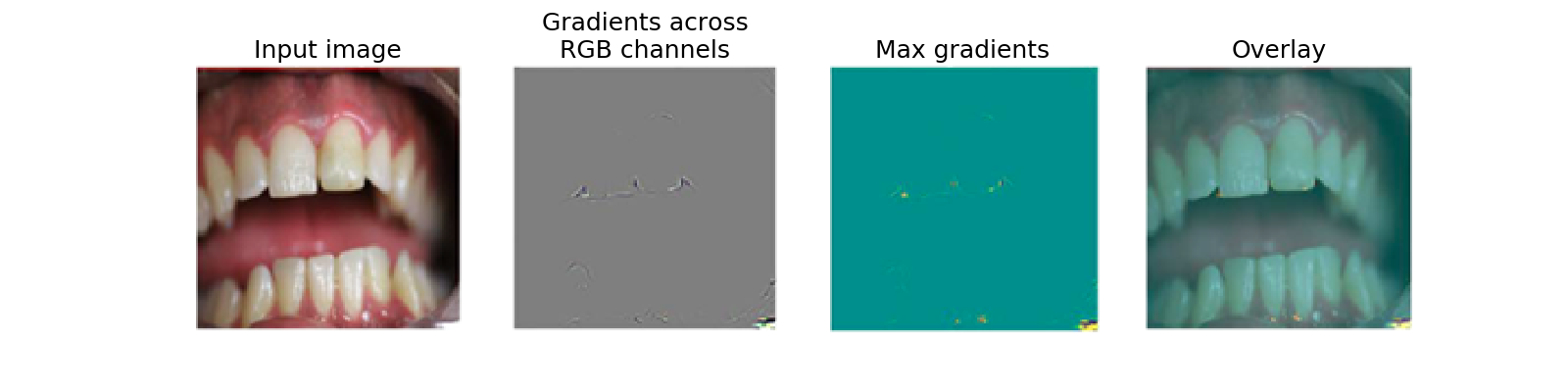


**Supplementary Figure 3: Saliency maps where activated regions are incorrect**

**a**. Rotation and displacement


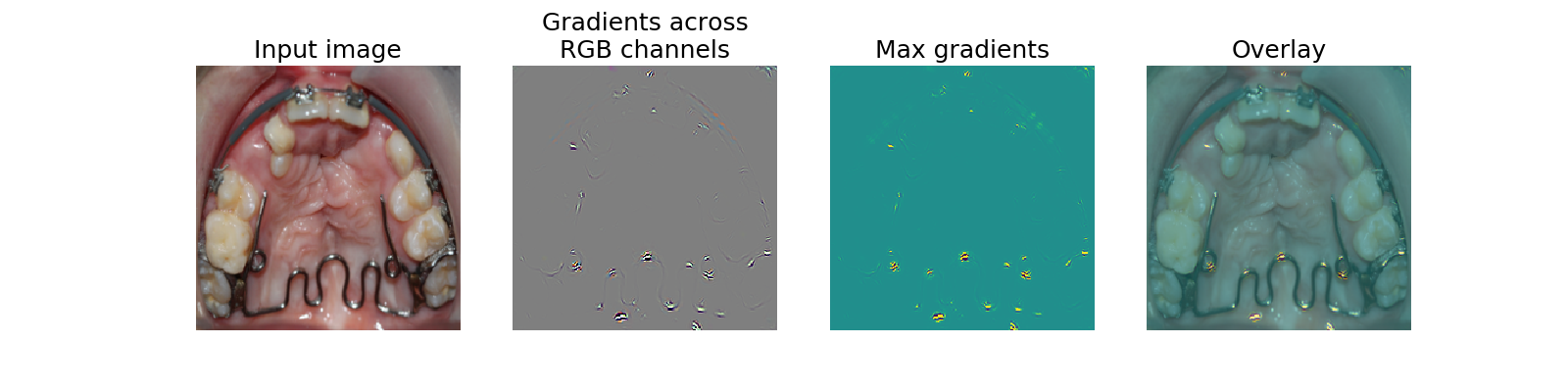


**b**. Mammalons


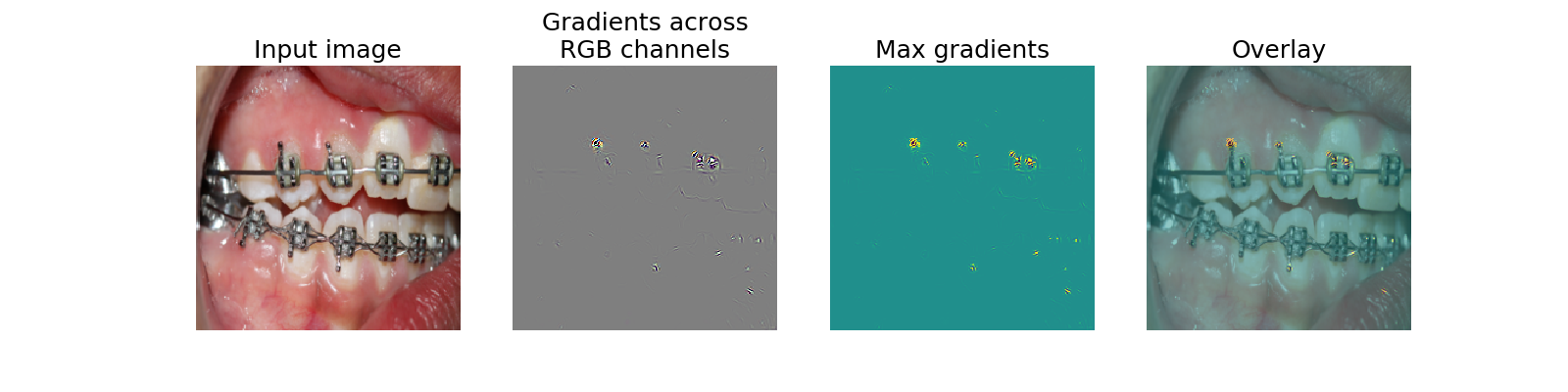


**c**. Mammalons
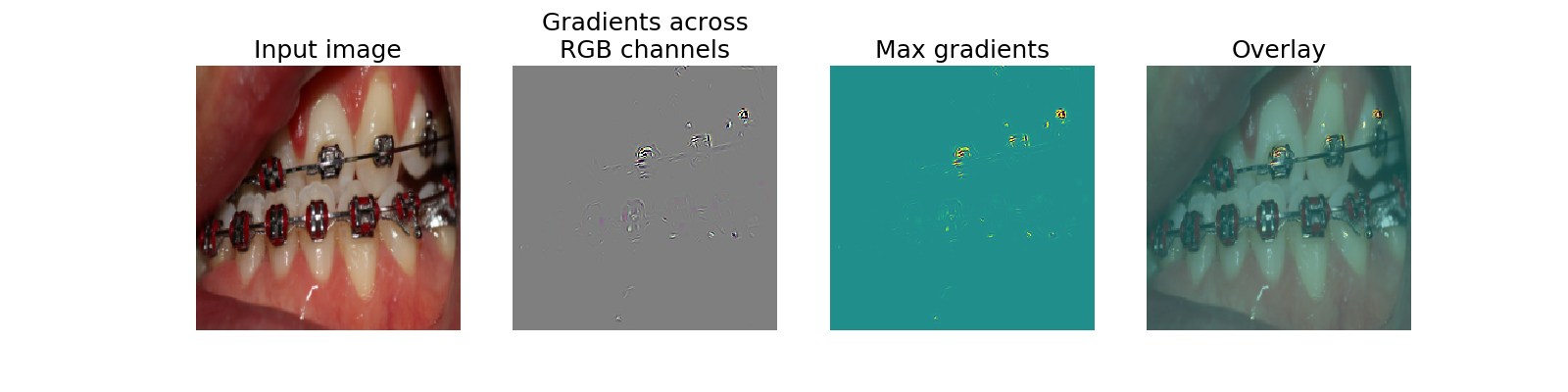


**d**. Displaced


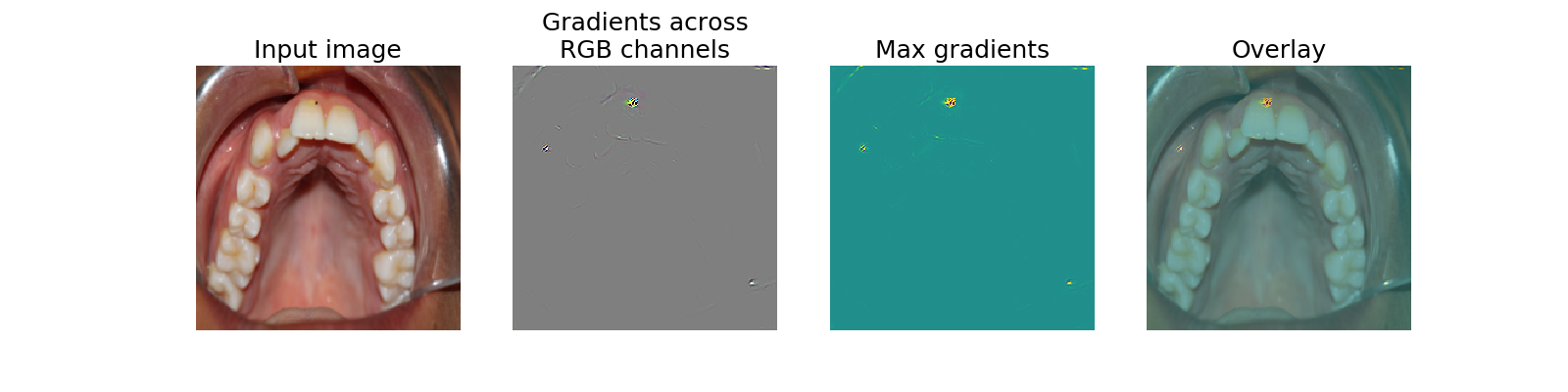


**e**. Hypocalcification
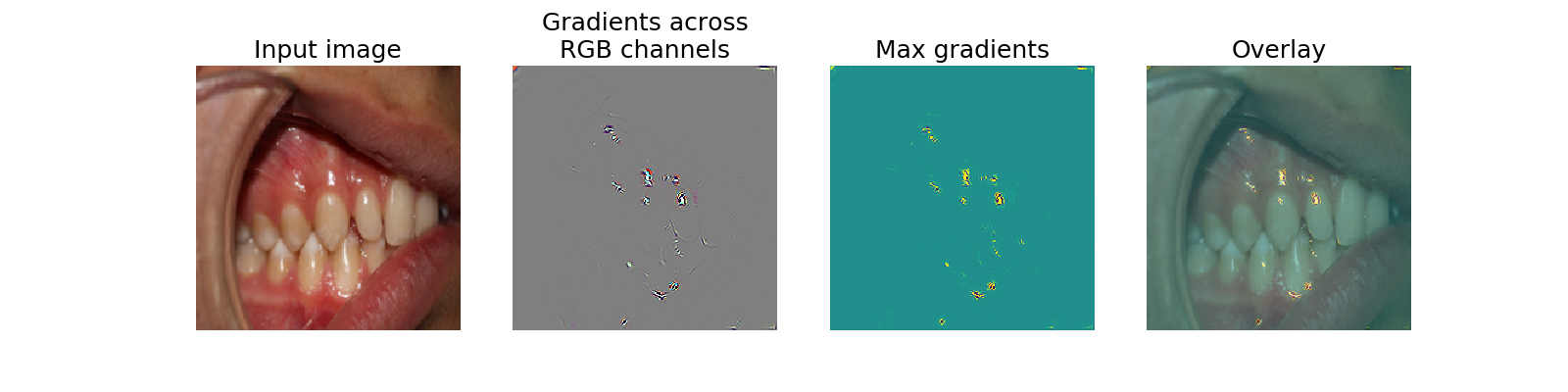


**f**. Microdontia


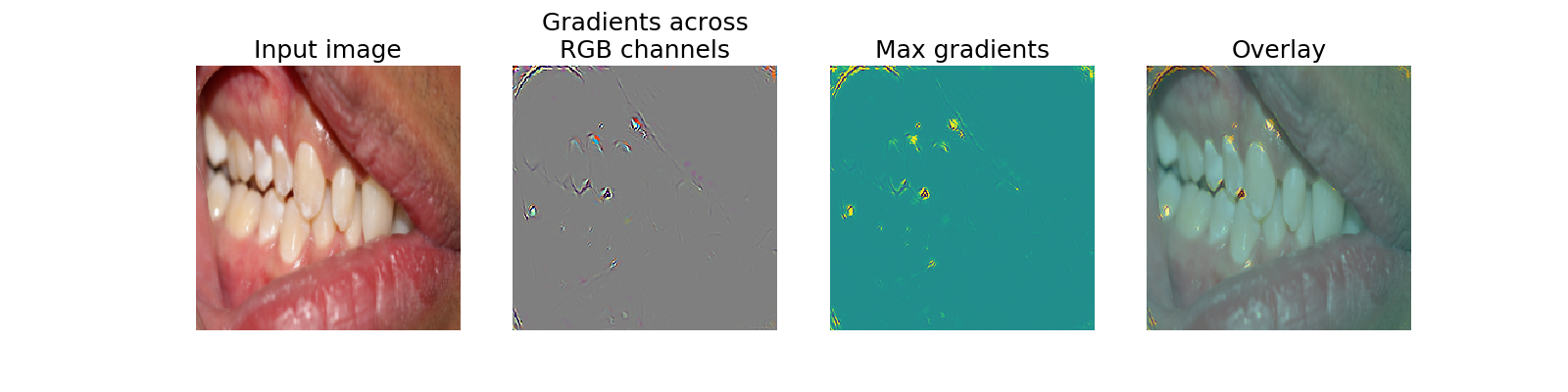


**g**. Agenesis
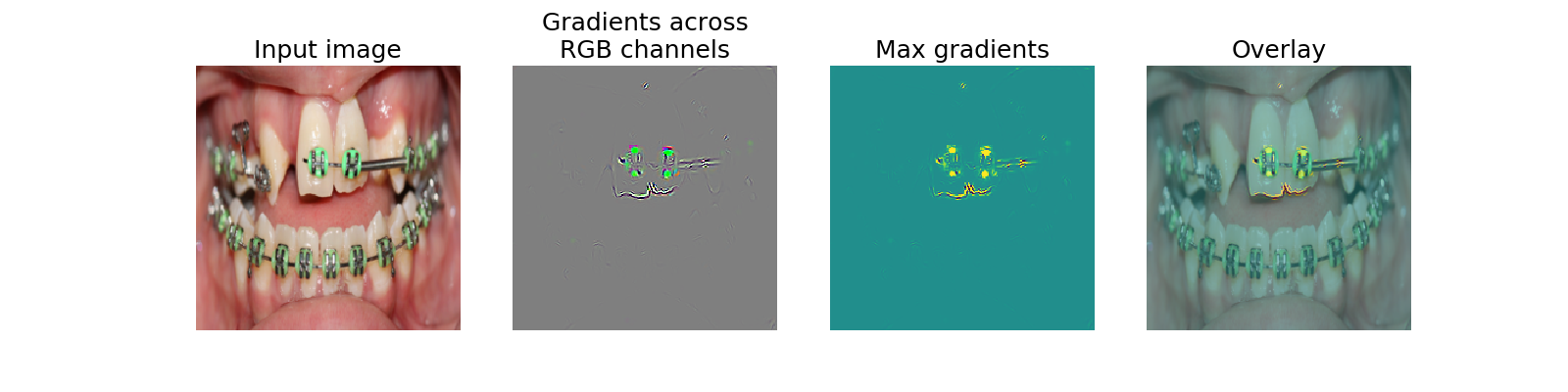


**h**. Supernumerary
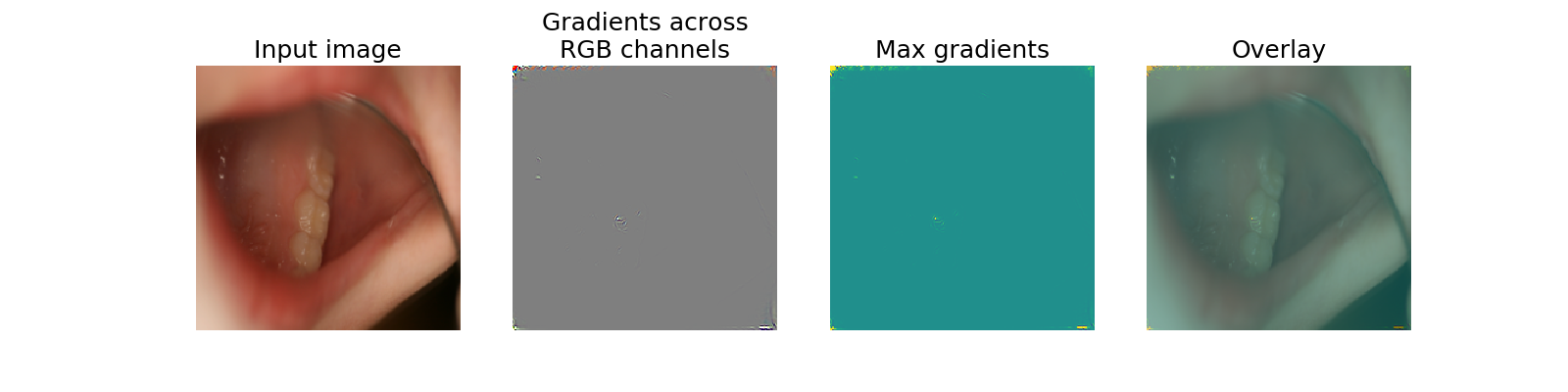


| Number of Anomalies per Image | Number of Images |
| --- | --- |
| 0 | 468 |
| 1 | 5257 |
| 2 | 12,167 |
| 3+ | 20,342 |

**Supplementary Table 1. Number of Anomalies Per Image**

Note: Number of photos with 0, 1, 2, or 3 or more anomalies.

Figure/Table Legend:

**Supplementary Figure 1. Intraoral Photograph Evaluation Form Example**

**Supplementary Figure 2: Examples of Correct Prediction by Model and not by Human**

Note: Overlay is the input image overlaid with the gradient images. These are representative examples of anomalies depicting what the algorithm sees.

**Supplementary Figure 3: Incorrect Activation Regions in Saliency Maps**

Subfigures a, b, c, and g are examples of when the model is distracted by dental appliances such as braces. Figure 3h is an example of when the model does not have strong activation regions in the mouth due to only a few teeth being shown.

**Supplementary Table 1. Number of Anomalies Per Image**

Note: Number of photos with 0, 1, 2, or 3 or more anomalies.
